# Computational Chemogenomics Drug Repositioning Strategy Enables the Discovery of Epirubicin as a New Repurposed Hit for Plasmodium falciparum and P. vivax

**DOI:** 10.1128/AAC.02041-19

**Published:** 2020-08-20

**Authors:** Letícia Tiburcio Ferreira, Juliana Rodrigues, Gustavo Capatti Cassiano, Tatyana Almeida Tavella, Kaira Cristina Peralis Tomaz, Djane Clarys Baia-da-Silva, Macejane Ferreira Souza, Marilia Nunes do Nascimento Lima, Melina Mottin, Ludimila Dias Almeida, Juliana Calit, Maria Carolina Silva de Barros Puça, Gisely Cardoso Melo, Daniel Youssef Bargieri, Stefanie Costa Pinto Lopes, Marcus Vinicius Guimarães Lacerda, Elizabeth Bilsland, Per Sunnerhagen, Bruno Junior Neves, Carolina Horta Andrade, Pedro Vitor Lemos Cravo, Fabio Trindade Maranhão Costa

**Affiliations:** a Laboratory of Tropical Diseases-Prof. Dr. Luiz Jacintho da Silva, Department of Genetics, Evolution, Microbiology and Immunology, University of Campinas-UNICAMP, Campinas, São Paulo, Brazil; b Laboratory of Molecular Modeling and Drug Design, LabMol, Faculdade de Farmácia, Universidade Federal de Goiás, Goiânia, Goiás, Brazil; c Global Health and Tropical Medicine (GHTM), Instituto de Higiene e Medicina Tropical, Universidade Nova de Lisboa, Lisbon, Portugal; d Instituto Leônidas e Maria Deane, Fundação Oswaldo Cruz–FIOCRUZ, Manaus, Amazonas, Brazil; e Fundação de Medicina Tropical-Dr. Heitor Vieira Dourado, Manaus, Amazonas, Brazil; f Synthetic Biology Laboratory, Department of Structural and Functional Biology, Institute of Biology, UNICAMP, Campinas, São Paulo, Brazil; g Department of Parasitology, Institute of Biomedical Sciences, University of São Paulo-USP, São Paulo, Brazil; h Department of Chemistry and Molecular Biology, University of Gothenburg, Gothenburg, Sweden; i LabChem–Laboratory of Cheminformatics, Centro Universitário de Anápolis–UniEVANGÉLICA, Anápolis, Goiás, Brazil

**Keywords:** DNA gyrase, chemogenomics, drug repositioning, epirubicin, malaria

## Abstract

Widespread resistance against antimalarial drugs thwarts current efforts for controlling the disease and urges the discovery of new effective treatments. Drug repositioning is increasingly becoming an attractive strategy since it can reduce costs, risks, and time-to-market. Herein, we have used this strategy to identify novel antimalarial hits. We used a comparative *in silico* chemogenomics approach to select Plasmodium falciparum and Plasmodium vivax proteins as potential drug targets and analyzed them using a computer-assisted drug repositioning pipeline to identify approved drugs with potential antimalarial activity. Among the seven drugs identified as promising antimalarial candidates, the anthracycline epirubicin was selected for further experimental validation. Epirubicin was shown to be potent *in vitro* against sensitive and multidrug-resistant P. falciparum strains and P. vivax field isolates in the nanomolar range, as well as being effective against an *in vivo* murine model of Plasmodium yoelii. Transmission-blocking activity was observed for epirubicin *in vitro* and *in vivo*. Finally, using yeast-based haploinsufficiency chemical genomic profiling, we aimed to get insights into the mechanism of action of epirubicin. Beyond the target predicted *in silico* (a DNA gyrase in the apicoplast), functional assays suggested a GlcNac-1-P-transferase (GPT) enzyme as a potential target. Docking calculations predicted the binding mode of epirubicin with DNA gyrase and GPT proteins. Epirubicin is originally an antitumoral agent and presents associated toxicity. However, its antiplasmodial activity against not only P. falciparum but also P. vivax in different stages of the parasite life cycle supports the use of this drug as a scaffold for hit-to-lead optimization in malaria drug discovery.

## Abstract (precis)

Widespread resistance against antimalarial drugs thwarts current efforts for controlling the disease and urges the discovery of new effective treatments. Drug repositioning is increasingly becoming an attractive strategy since it can reduce costs, risks, and time-to-market. Herein, we have used this strategy to identify novel antimalarial hits. We used a comparative *in silico* chemogenomics approach to select Plasmodium falciparum and Plasmodium vivax proteins as potential drug targets and analyzed them using a computer-assisted drug repositioning pipeline to identify approved drugs with potential antimalarial activity.

## INTRODUCTION

Malaria is an infectious disease caused by protozoan parasites from the *Plasmodium* genus that are transmitted by the bite of *Anopheles* mosquitoes. Among the five malaria parasite species capable of infecting humans, Plasmodium falciparum and Plasmodium vivax are in the spotlight due to their wide geographic distribution and increasing morbidity and mortality rates. With an estimated 228 million cases reported in 2018 (1), malaria still imposes a heavy burden upon tropical and subtropical areas of the globe, accounting for more than 400,000 deaths annually. Moreover, although P. vivax causes fewer fatalities, it has a wider geographic distribution with a larger number of people at risk of infection.

In the absence of an effective vaccine against malaria, chemotherapy constitutes the main approach toward disease control. The introduction of artemisinin combined therapies (ACTs) has widely contributed to reducing malaria-associated incidence and mortality (2). However, malaria parasites have evolved resistance to available antimalarial drugs. Even ACT resistance has been increasingly observed in the form of delayed parasite clearance (3), menacing control efforts. One of the underlying phenomena contributing to the sudden decline of antimalarial efficacy is cross-resistance, i.e., resistance to an antimalarial drug may facilitate the evolution of resistance against compounds with similar molecular mechanisms (4). Hence, new antimalarial drugs with novel molecular targets and distinct modes of action are urgently needed.

Malaria drug discovery efforts traditionally focus on P. falciparum due both to its high mortality rates and the availability of continuous *in vitro* culture. However, severe P. vivax cases occur (5) and could be linked to chloroquine resistance (6), which has been reported worldwide, therefore highlighting the need for identifying novel anti-P. vivax drugs. The lack of a continuous *in vitro* culture system and the rapid onset of circulating gametocytes in the peripheral blood upon infection (7) represent major bottlenecks in fighting vivax malaria, reinforcing the need to look beyond the blood stage for drug development and consequently disease control and/or elimination.

The drug development process is long, costly, and very prone to failure. Thus, it is crucial to advance strategies to improve the success rate in malaria drug research and development (R&D). Drug repositioning is one such strategy (8) that resorts to approved and marketed drugs as starting points for the development of new therapies. As all approved drugs have detailed information on their pharmacokinetic and safety profiles, when a new application for a drug is identified, the molecule can be rapidly advanced into clinical trials. Although few drugs have been repurposed to treat malaria (9, 10), most have resulted from either serendipity or phenotypical screening of approved drug libraries (11, 12), with little prior evidence-based data to support their efficacy. Consequently, such approaches can be time-consuming and expensive. In a bid to boost drug repositioning efforts, rational-based approaches based on *in silico* evaluations allow molecule efficacy predictions, narrowing down the panel of drugs for experimental evaluation (13).

We have previously developed an *in silico* target-based chemogenomics drug repurposing workflow (14), which led us to identify approved drugs with confirmed activity against the P. falciparum apicoplast (15), multiple life cycle stages of Schistosoma mansoni (16, 17), paracoccidioidomycosis (18), *Schistosoma* sp. protein kinases (19), and *Leishmania* sp. kinases (20). In the present work, we used a comparative phylogenomics approach in order to identify predicted targets of P. falciparum and P. vivax, which are present in the parasite’s proteome but expected to be absent in humans. Among a list of approved drugs against these targets, we identified a promising antimalarial hit, epirubicin, an approved anthracycline, which is used for chemotherapy to treat breast cancer (21). Driven by encouraging results obtained from preliminary *in vitro* phenotypic assays in P. falciparum asexual stages, we sought to explore the antiplasmodial potential of epirubicin throughout the parasite life cycle and carried out functional investigations to explore its mechanism of action.

## RESULTS

### Computational chemogenomics screening.

**FIG 1:**
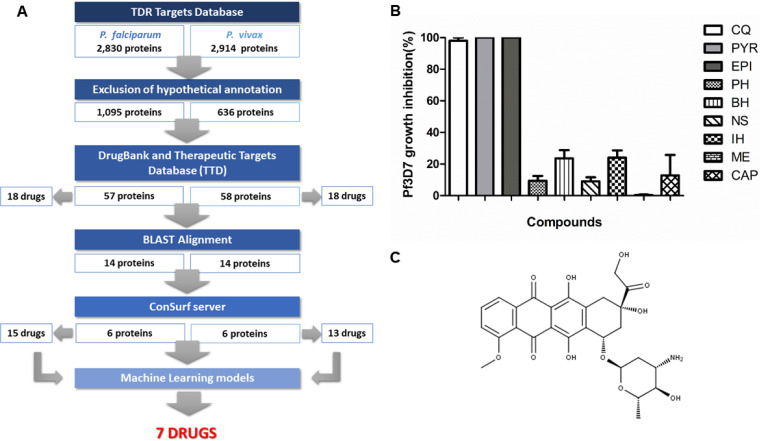
*In silico* chemogenomics strategy for drug repurposing. (A) Flowchart summarizing the *in silico* repositioning chemogenomics strategy and corresponding results. (B) Phenotypic screening for inhibition of P. falciparum 3D7 growth *in vitro* using compound candidates for repositioning at 5 μM. (C) Chemical structure of epirubicin. CQ, chloroquine; PYR, pyrimethamine; EPI, epirubicin; PH, phenoxybenzamine hydrochloride; BH, besifloxacin hydrochloride; NS, nedocromil sodium; IH, isoproterenol hydrochloride; ME, mesalazine; CAP, captopril.

We developed a computational chemogenomics workflow (Fig. 1A) for drug repurposing for malaria. Initially, we carried out a genome-wide phylogenomics analysis in the TDR Targets Database to prioritize P. falciparum and P. vivax proteins without predicted orthologues in humans and rodents. Consequently, we obtained a list of 2,830 and 2,914 P. falciparum and P. vivax proteins, respectively. We further filtered these proteins by removing from the list all peptides that were annotated as hypothetical, resulting in a panel of 1,095 and 636 proteins for P. falciparum and P. vivax, respectively.

Subsequently, putative antiplasmodial drugs were screened using the underlying assumption that homologous proteins have an enhanced probability of sharing the same ligands (14, 17). A sequence-based similarity search was performed between *Plasmodium* proteins and all drug targets available in DrugBank (22) and the Therapeutic Targets Database (TTD) (23, 24), which provide detailed information about drugs and their targets. This strategy resulted in a list of 115 potential *Plasmodium* targets (i.e., 57 and 58 confirmed targets for P. falciparum and P. vivax, respectively) that could interact with 36 approved drugs (18 for P. falciparum and 18 P. vivax).

In order to increase the confidence in drug-target predictions, we investigated the conservation of functional regions of prioritized *Plasmodium* proteins and their drug target homologues. This strategy allowed us to prioritize *Plasmodium* proteins with conserved binding sites, thereby increasing the probability that each of the drugs bears biological activity. Consequently, we selected 28 approved drugs (15 for P. falciparum and 13 for P. vivax) that could potentially interact with 12 potential druggable *Plasmodium* targets for further analysis. A bibliographic search allowed us to identify which among the selected drugs had not been tested or proposed as antimalarial before, leading to a final list of 12 approved drugs. Detailed information about the list of predicted drugs and their associated *Plasmodium* targets are provided in Table S1 in the supplemental material.

### Machine learning models.

Machine learning (ML) models were built to prioritize which drugs would be experimentally evaluated by distinguishing between active versus inactive drugs against blood stages of P. falciparum 3D7 (susceptible to chloroquine) and W2 (multidrug resistant) strains. Initially, two data sets with phenotypic bioassay data for 3D7 (susceptible to chloroquine) (25–30) and W2 (resistant to chloroquine) (26, 28, 31–35) strains of P. falciparum were compiled from the PubChem BioAssay database (36, 37). Subsequently, all chemical structures and corresponding biological information were carefully curated according to the protocols proposed by Fourches and colleagues (38–40). Furthermore, ML models were trained by combining Avalon fingerprints (generated from chemical structures) and the Gradient Boosting Machine (GBM) algorithm (41). According to the statistical results of a 5-fold external cross-validation procedure (see Materials and Methods), the model developed for the prediction of antiplasmodial activity against the 3D7 strain showed a correct classification rate (CCR) of 74%, sensitivity (SE) of 73%, specificity (SP) of 74%, and coverage of 70%. The model developed for the W2 strain showed a CCR of 71%, SE of 71%, SP of 72%, and coverage of 70%. The 12 drugs prioritized from computational chemogenomics analysis were submitted to ML models for prediction of antiplasmodial activity. The predictions were considered reliable when they were within the chemical space (i.e., applicability domain) of the training set compounds used to build the ML models. At the end of this process, seven drugs (epirubicin, phenoxybenzamine, besifloxacin, nedocromil, isoprenaline, mesalazine, and captopril) with potential antiplasmodial activity were selected for biological evaluation.

### *In vitro* phenotypic screening of selected drugs.

We carried out a primary *in vitro* screen to determine the ability of each drug to inhibit the growth of the chloroquine-sensitive P. falciparum 3D7 strain at 5 μM (Fig. 1B). The drugs used from the list of potential candidates from the *in silico* approach were tested in parallel with chloroquine and pyrimethamine as controls. These results showed that epirubicin (Fig. 1C), which we predicted to inhibit P. falciparum DNA gyrase subunit A, was capable of inhibiting P. falciparum 3D7 growth at nearly 100%, and therefore, we decided to investigate its antimalarial activity against other parasite life cycle stages and species and to determine its possible mode of action.

### Epirubicin is effective *in vitro* against *Plasmodium* asexual stages in the nanomolar range.

**FIG 2:**
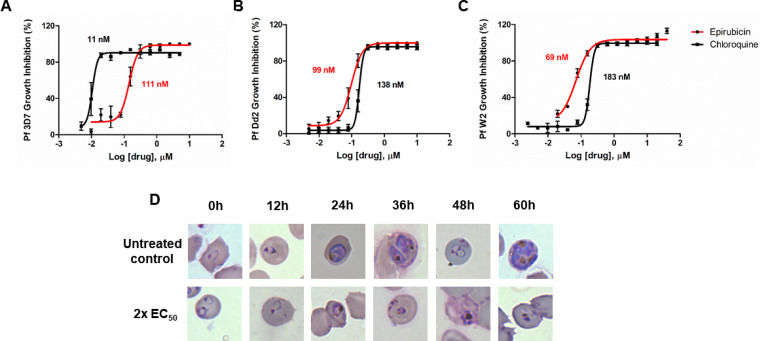
*In vitro* antimalarial activity of epirubicin against P. falciparum strains. Inhibition curves for epirubicin *in vitro* against chloroquine-sensitive (3D7) (A) and multidrug-resistant (Dd2 and W2) (B, C) P. falciparum strains. Data are derived from three independent experiments. (D) Microscopy of Giemsa-stained thin smears of highly synchronized parasite cultures starting at the ring stage (1% parasitemia). First line shows parasites treated with DMSO (control), while second line shows culture continuously treated with epirubicin at concentrations 2-fold the EC_50_ value. Images were collected using an Olympus microscope equipped with 100× lens objective and a camera.

Considering that epirubicin cleared 100% of parasites at 5 μM, we sought to determine its 50% effective concentration (EC_50_). The drug was tested in serially diluted concentrations against drug-sensitive P. falciparum 3D7 and multidrug-resistant W2 and Dd2 strains, with chloroquine as a control. We found that epirubicin has a modest effect against the drug-sensitive strain P. falciparum 3D7 compared with chloroquine (around 10-fold less active), but the EC_50_ just over 100 nM (110.7 ± 22.4 nM) places it in the medium nanomolar range (Table 1; Fig. 2A). Importantly, the drug showed similar or even greater potency against drug-resistant strains Dd2 and W2 (EC_50_ of 99.5 ± 25.1 and 68.9 ± 5.1 nM, respectively) (Fig. 2B and C).

**TABLE 1:** *In vitro* antiplasmodial activity and cytotoxicity of epirubicin^*a*^

Drug	EC_50_ (nM) of P. falciparum strain:			COS-7		HepG2	
	3D7	Dd2	W2	CC_50_ (nM)	SI	CC_50_ (nM)	SI
Epirubicin	110.7 ± 22.4	99.5 ± 25.1	68.9 ± 5.1	5,480 ± 720	49.4	80 ± 30	0.7
Chloroquine	11.3 ± 3.6	137.8 ± 34.1	182.8 ± 11.5	ND	ND	ND	ND

a EC_50_, half of the maximal inhibitory concentration in P. falciparum; CC_50_, half of the maximal cytotoxic concentration in mammalian cells; SI, selectivity index calculated from CC_50_/EC_50_ (3D7); ND, not determined. Data are means ± SD and are derived from three independent experiments.

In a bid to further understand the antimalarial activity of epirubicin, we followed epirubicin-treated and untreated P. falciparum cultures for 60 h in order to evaluate morphological changes in the parasites (Fig. 2D). Epirubicin showed activity toward the early trophozoite stage since morphological deformities could be observed only 24 h after incubation. The treated parasites were not viable since no ring- or trophozoite-stage parasites were observed when the culture was continued to the 48 h or 60 h, respectively.

We also evaluated the cytotoxicity of epirubicin *in vitro* using two mammalian cell lines. A nontumoral mammalian cell line (COS7) was shown to be moderately sensitive to epirubicin, presenting half maximal cytotoxic concentration (CC_50_) around 5 μM (5.48 ± 0.72 μM). Predictably, we found epirubicin to be significantly toxic to tumoral HepG2 cells (0.08 ± 0.03 μM), with 70-fold more sensitivity than COS7 cells. Considering the CC_50_ of epirubicin in COS7 cells, we calculated its selectivity index around 49 (Table 1).

### Epirubicin mitigates mice parasitemia *in vivo* but does not resolve the infection.

After demonstrating the activity of epirubicin activity against P. falciparum *in vitro*, we investigated whether this activity is sustained in an *in vivo* model of infection. Plasmodium yoelii 17XNL-infected mice were treated with epirubicin for 4 days (days 0 to 3 postinfection [p.i.]) (see Table S2 in the supplemental material). As shown in Table 2, treating animals with 2 mg/kg of body weight showed no potency on inhibiting parasitemia. The intermediate dose of 4 mg/kg showed 82% of inhibition on day 3 p.i., but on day 5 p.i., this inhibition dropped below 20%. However, 6 mg/kg of epirubicin significantly reduced parasitemia compared with the untreated control by 95% and 78% on day 3 p.i. and at day 5 p.i. (48 h after the end of treatment). On day 7 p.i., the highest dose tested reached about 50% of parasite growth inhibition.

**TABLE 2:** *In vivo* parasitemia inhibition of P. yoelii 17XNL-infected mice treated with epirubicin for 4 days

Group	Mean percentage parasitemia ± SD (% inhibition^*a*^ of parasite growth)		
	Day 3	Day 5	Day 7
Nontreated	1.3 ± 0.8	1.7 ± 0.4	3.6 ± 1.5
Treated with epirubicin (mg/kg)			
2	0.6 ± 0.4 (56)	2.2 ± 1.5 (0)	6.9 ± 5.3 (0)
4	0.2 ± 0.2 (82)	1.4 ± 1.2 (19)	7.5 ± 6.1 (0)
6	0.1 ± 0.1 (95)	0.4 ± 0.3 (78)	1.8 ± 1.3 (51)

a Values are expressed as mean percentage parasitemia inhibition relative to nontreated control. Data are means ± SD and are derived from three independent experiments (control, *n* = 18; 2 mg/kg, *n* = 16; 4 mg/kg, *n* = 15; 6 mg/kg, *n* = 16).

### Epirubicin inhibits parasite maturation of P. vivax clinical isolates.

**FIG 3:**
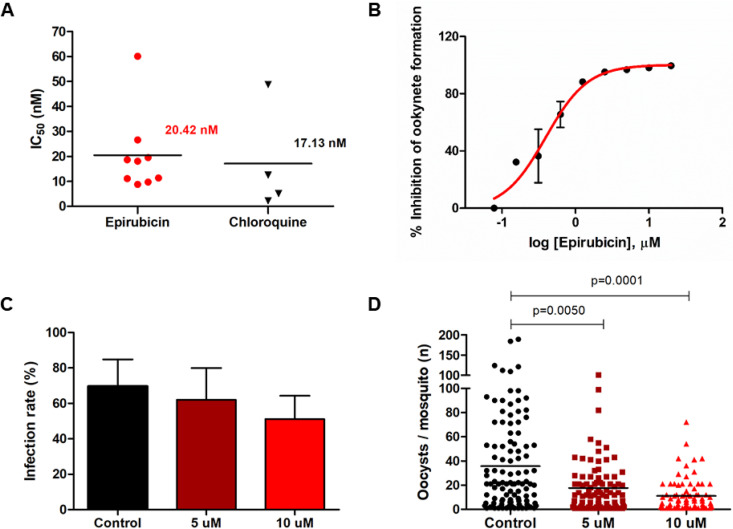
Blood stage and transmission-blocking activity of epirubicin on P. vivax. (A) *Ex vivo* drug susceptibility of epirubicin in clinical isolates of P. vivax from Manaus, Brazil. (B) *In vitro* inhibition of P. berghei gamete fertilization and ookinete conversion by epirubicin on conversion assay. Infection rates (C) and oocyst intensity (D) in membrane feeding assay (MFA) using *An. aquasalis* mosquitoes exposed to P. vivax-infected blood. For membrane feeding assays, seven biological replicates were performed.

The *ex vivo* inhibition potential of epirubicin was evaluated in P. vivax isolates by the schizont maturation assay using 9 clinical isolates from P. vivax-infected patients. Blood samples used for this purpose showed an initial parasitemia of 5,407.33 ± 2,372.21 parasites/μl with 65.67% (ranging from 40% to 88%) of the observed parasites in the ring stage. The *ex vivo* activity of epirubicin was tested in all 9 isolates, whereas chloroquine activity was evaluated in parallel in 4 of them (Fig. 3A). Epirubicin and chloroquine EC_50_s were 20.42 nM (range, 8.76 to 60.05 nM) and 17.13 nM (range, 2.16 to 48.76 nM), respectively. No significant difference was observed between the compound inhibition potencies (*P* = 0.4140; Mann-Whitney test).

### Epirubicin blocks transmission by inhibiting Plasmodium berghei ookinete development *in vitro*.

A conversion assay using sexual stages of P. berghei with ability to fertilize *in vitro* was conducted to examine the transmission blocking potential of epirubicin. Epirubicin showed activity in a dose-response manner, inhibiting approximately 80% of ookinete development at 1 μM with an EC_50_ of 0.39 μM (Fig. 3B). This result suggests that epirubicin interferes in fertilization, sexual recombination, and consequent ookinete development in P. berghei.

### Epirubicin blocks *in vivo* parasite transmission in P. vivax-infected *Anopheles* mosquitoes.

Once the transmission blocking activity of epirubicin was observed *in vitro*, we aimed to evaluate its activity *in vivo* for the inhibition of mosquito infection using membrane feeding assays. For this purpose, seven clinical samples of P. vivax were used. The prevalence of oocysts in the mosquitoes was not significantly reduced in the presence of epirubicin (*P* = 0.1636; analysis of variance [ANOVA]-Dunnet’s) (Fig. 3C). Among infected mosquitoes, epirubicin led to a dose-dependent decrease in the absolute number of oocysts found in mosquitoes midguts. While the control group presented a mean of 23.83 oocysts per mosquito, the groups treated with 5 and 10 μM epirubicin presented 11.22 and 5.54, respectively, i.e., a decrease of 53% and 76% in oocyst intensity (Fig. 3D).

### Chemical genomic profiling identifies protein glycosylation affected by epirubicin.

**FIG 4:**
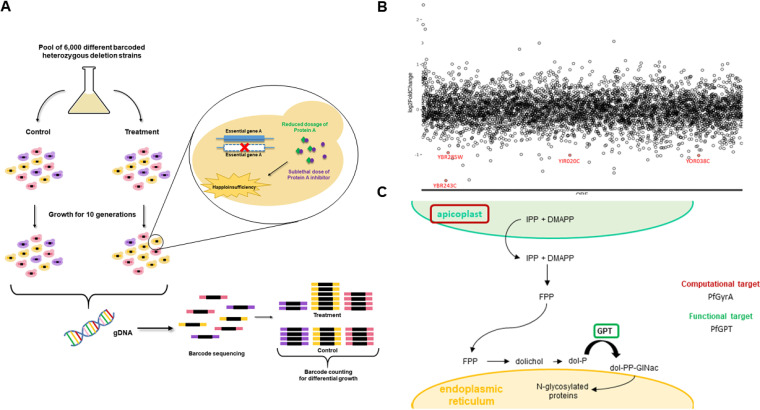
Investigation of the mechanism of action of epirubicin. (A) Chemical genomic profiling assay. A pool of ∼6,000 barcoded heterozygous strains deleted for each gene of the S. cerevisiae genome is cultivated in the presence and absence of a sublethal dose of epirubicin. Yeast culture is diluted 1:20 at 5 generations, and growth is allowed until 10 generations. Genomic DNA is collected for sequencing. Barcode counting allows identification of strains depleted under treatment, suggesting hypersensitivity to epirubicin. (B) Drug-induced haploinsufficiency profiling of mutant haploid heterozygous S. cerevisiae upon treatment with epirubicin EC_20_. Log fold change is plotted on the *y* axis as a function of depleted yeast strains alphabetically ordered by their respective open reading frame (ORF). The lower the log fold change value, the more sensitive the strain is. Red dots highlighted in the chart represent the ORFs that meet the conditions *P* value of <0.001 and log_2_ fold change of <0. (C) Scheme summarizing the proposed mechanism for the mode of action of epirubicin in the malaria parasite. Abbreviations: gDNA, genomic DNA; IPP, isopentenyl pyrophosphate; DMAPP, dimethylallyl pyrophosphate; FPP, farnesyl pyrophosphate; dol-P, dolichol phosphate; GPT, dolichol phosphate *N*-acetylglucosamine-1-phosphotransferase; dol-PP-GlcNAc, dolichol pyrophosphate *N*-acetylglucosamine.

The first steps in investigating the mechanism of action of epirubicin were taken with the chemical genomic profiling assay, based on the premise that diploid yeast hemizygous for a gene is hypersensitive to an inhibitor drug directed to the product of that gene (Fig. 4A). Epirubicin inhibition assays performed in wild-type yeast revealed an EC_20_ of 2.9 μM (data not shown), which was the drug concentration used to perform the chemical genomic profiling competition assays. Pools of approximately 6,000 heterozygous yeast strains were grown for 10 generations under treatment conditions. Analysis of the haploinsufficiency profile of heterozygous yeast strains allowed the identification of epirubicin-sensitive strains (see Table S3 in the supplemental material), four of which presented statistical significance in their depletion compared with the control (Fig. 4B). A mutant strain that has a reduced level of an essential gene involved in protein N-glycosylation, *Δalg7*, which codes for the dolichol phosphate *N-acetylglucosamine*-1-phosphotransferase (GPT) (42), was found among the hits, which indicates that protein glycosylation can be potentially affected by epirubicin treatment.

Considering that P. falciparum has a GPT orthologue present in its genome (43), we suggest that epirubicin could interfere with isoprenoid metabolism in the malaria parasite, either via apicoplast disruption or GPT enzyme inhibition (Fig. 4C).

### Molecular modeling and docking studies of epirubicin with its two predicted targets.

Considering the predicted and functional molecular targets identified for epirubicin, we sought to explore its potential to bind to each of the proposed targets. We built the P. falciparum DNA gyrase (*Pf*GyrA) and dolichol phosphate *N-acetylglucosamine*-1-phosphotransferase (*Pf*GPT) structures through a homology modeling approach. The DNA gyrase model was based on PDB ID 2XKJ from Acinetobacter baumannii (44), which had 30.04% sequence identity with *Pf*GyrA. This model was analyzed at MolProbity server and showed a MolProbity score of 1.83, reflecting in a single score the crystallographic resolution at which those values would be expected (45) (see Table S4 in the supplemental material). Also, the Ramachandran plot of the DNA gyrase model showed that 96.96% of the residues lie in the most favorable regions (Table S4; see Fig. S1 in the supplemental material). The *Pf*GPT model was based on PDB ID 6BW5 from Homo sapiens (46) which had 45.27% sequence identity with P. falciparum GPT. The *Pf*GPT model showed 97.84% of their residues in the most favorable regions of the Ramachandran plot and MolProbity score of 1.93 (see Table S5 in the supplemental material; see Fig. S2 in the supplemental material).

**FIG 5:**
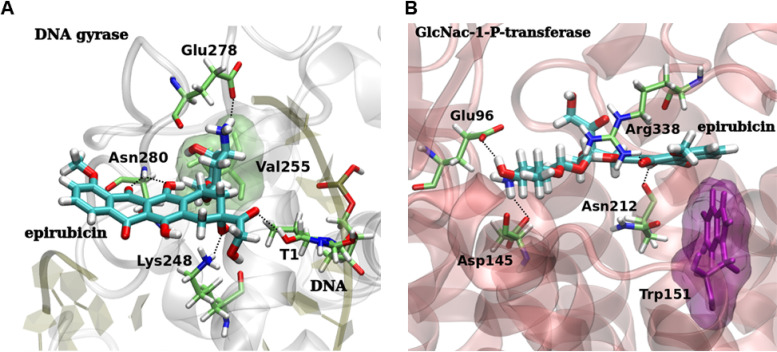
3D intermolecular interactions obtained by molecular docking between epirubicin and its two potential targets. (A) Epirubicin (carbon atoms in cyan) docked within DNA binding site of P. falciparum gyrase-DNA (docking score, −7.96 kcal·mol^−1^), highlighting the main hydrogen bonds (dotted black lines) within epirubicin and the amino acid residues Lys248, Glu278, and Asn280, as well as the interaction with the DNA base pair thymine (T1). Epirubicin also made hydrophobic interactions with the Val255 residue (transparent green surface). (B) Docked pose of epirubicin with P. falciparum GPT protein (docking score, −7.92 kcal·mol^−1^), highlighting the main hydrogen bonds (dotted black lines) with the amino acid residues Glu96, Asp145, Asn212, and Arg338. Epirubicin was also able to make a *T-stacking* interaction with the Trp151 residue (transparent purple surface). Amino acids are colored as carbon atoms in green, oxygen in red, hydrogen in white, and nitrogen in blue.

We then performed molecular docking calculations in order to investigate the binding mode and to shed some light into the affinity of epirubicin against the two targets. Epirubicin docked in *Pf*GyrA (Fig. 5A) and *Pf*GPT (Fig. 5B) presented good binding affinities, with docking scores of −7.96 and −7.92 kcal·mol^−1^, respectively. Moreover, docking calculations of epirubicin with a *Pf*GyrA structure with a DNA strand and without a DNA strand (data not shown) reinforce the fact that epirubicin could act as a DNA intercalating agent since it has better docking scores in gyrase-DNA (−7.96 kcal·mol^−1^) than gyrase without a DNA strand (−6.38 kcal·mol^−1^).

## DISCUSSION

We have used combined chemogenomics, bioinformatics, and cheminformatics approaches in an attempt to identify approved drugs as new potential hits against P. falciparum and P. vivax, the two most important of the human malaria parasites. In doing so, we employed a drug repurposing strategy, which allows the identification of new applications for drugs that have already been approved for use in humans. Consequently, we obtained a final list of seven drugs that were expected to be active but had never been tested as antimalarials previously, especially against P. vivax. These drugs are anticipated to inhibit different parasitic targets and span several different original indications, including antiviral, anticancer, and antibiotics (Table S1). Thereafter, we proceeded to test their *in vitro* ability to inhibit the growth of the drug-sensitive strain P. falciparum 3D7. Epirubicin was shown to be active, with an EC_50_ at the nanomolar level (111 nM, 99 nM, and 69 nM for P. falciparum 3D7, Dd2, and W2 strains). Previously, Mogire and coworkers also identified epirubicin *in silico* as a potential candidate for drug repurposing against P. falciparum, but they did not test it experimentally (47).

Epirubicin is an anthracycline which is the 4′-epi-isomer of doxorubicin used in combination with other drugs for treating breast cancer. The compound exerts its antitumoral effects by interfering with DNA synthesis and function and has reported activity over mammalian cells (48). Epirubicin inhibits nucleic acid and protein biosynthesis by intercalation between base pairs, forming an epirubicin-DNA complex that inhibits topoisomerase II activity. Its interference with both DNA replication and transcription is also conferred by inhibition of DNA helicase activity (48).

In the present study, *in vitro* dose-response experiments to calculate the EC_50_ of epirubicin showed significant activity against both sensitive and multidrug-resistant P. falciparum strains. This is an important observation, as the great majority of the parasite population in natural settings is nowadays highly resistant to most drugs worldwide, including chloroquine. For instance, in Brazil, an area of chloroquine resistance, the mean parasite EC_50_ among P. falciparum Amazonian isolates has been shown to be nearly 300 nM (49), which is nearly 4-fold higher than the activity we reported here for epirubicin in resistant P. falciparum strain W2. We also show that epirubicin is equally effective against both chloroquine-sensitive and chloroquine-resistant parasites.

A lack of cross-resistance of epirubicin with clinically used antimalarial drugs is likely due to its predicted target, the DNA gyrase subunit A, involved in the process of DNA synthesis in the malarial apicoplast, while the targets of chloroquine are related to inhibition of heme polymerization in the parasite’s food vacuole (50). Consequently, because the predicted targets and their respective localization within the cell are very distinct, the phenomenon of cross-resistance between the two drugs is highly unlikely. This phenotype of epirubicin’s activity against a chloroquine-resistant strain was corroborated *in vivo* in a nonlethal P. yoelii 17X chloroquine-resistant strain (51).

We also found that epirubicin is capable of inhibiting the maturation of blood-stage parasites in samples from P. vivax clinical isolates from Manaus, Brazil. Evaluating the drug’s potency against P. vivax is especially relevant in the context of chloroquine resistance (52), the golden standard antimalarial drug for malaria vivax treatment in regions of endemicity for over 60 years (53), and considering that chloroquine (CQ) resistance in P. vivax has been linked to increasing numbers of reports of infection severity (54).

In addition to activity against asexual blood-stage parasites, epirubicin also was shown to inhibit gamete fertilization and consequent ookinete formation in P. berghei and in P. vivax. When assessing the *in vivo* transmission blocking activity through a membrane feeding assay, epirubicin was able to reduce the number of P. vivax oocysts found in the midguts of infected mosquitoes. Many features of P. vivax biology have direct impact on its pathogenesis (7, 55) and render infected patients efficient infection reservoirs. To the best of our knowledge, no study so far has reported the antimalarial potential of anthracyclines, such as epirubicin, against P. vivax asexual and sexual stages. As such, the results presented here also pave the way for the optimization of anthracyclines as a new class of compounds to treat vivax malaria.

The first experimental steps in elucidating the possible mechanism of action of epirubicin were taken using the haploinsufficiency chemical genomic profiling assay, an approach based on the premise that yeast containing a gene expressed in heterozygosity will become hypersensitive to an inhibitor compound directed to the product of that gene. By exploiting a collection of mutant yeast strains, this methodology allows us to explore biochemical pathways and molecular targets affected by a compound at the genomic level. Since the therapeutic target predicted by computational screening for epirubicin in *Plasmodium* sp. is the subunit A of the apicoplast DNA gyrase (derived from a prokaryotic plasmid) (56), we expected to find the depletion of the strain mutant for the *TOP2* gene, which encodes DNA topoisomerase II, an yeast enzyme structural and phylogenetically related to apicoplast gyrase (57). Unfortunately, strains heterozygous for *TOP2* were underrepresented in the initial strain pool, not allowing statistical evaluation of the sensitivity of this strain under epirubicin treatment and functionally validating P. falciparum DNA gyrase (*Pf*GyrA) as an epirubicin target.

The molecular target identified, Alg7, is essential in yeast and conserved among eukaryotes (58). Alg7 is the enzyme Dol-P-dependent GlcNAc-1-P transferase (GPT) (42), which participates in the initial stages of *N*-glycoprotein synthesis (59). P. falciparum has an Alg7 ortholog (60, 61) whose product is responsible for the synthesis of glycans bound to dolichol (58), a class of isoprenoids that participate in the biosynthesis of *N*-linked oligosaccharides (62). As it has been shown that the essential role of the *Plasmodium* apicoplast in the blood stage is the production of isoprenoid precursors (63), disruption of apicoplast function by epirubicin leads to the inhibition of dolichol biosynthesis and thereby protein *N*-glycosylation. Additionally, resistance to epirubicin in cancer cells can occur mediated by WNT glycoproteins, which are glycosylated by GPT (64). In this way, chemical genomic profiling results support our *in silico* prediction of epirubicin action upon the parasite apicoplast. Moreover, our docking calculations showed that epirubicin binds with similar affinity to both *Pf*GyrA and GPT, which indicates that its action upon protein glycosylation might not only be a consequence of apicoplast disruption but also a direct target.

In order to meet the need of a desirable drug candidate for malaria treatment, Target Product Profiles (TPPs) have been described (65, 66). Target Product Profiles comprise the ideal final products for antimalarial combinations, while candidate molecules for these formulations represent target candidate profiles. For malaria treatment, TPP-1 is intended for the management of acute uncomplicated malaria cases and TPP-2 for chemoprotection. However, epirubicin does not meet the requirements within the scope of the TPPs proposed, like rapid onset of action, dosing regimen, safety, and tolerability (66). This is most likely due to its narrow therapeutic window, as reported in this study, and parenteral administration (67), together with the widely reported cardiotoxicity associated with epirubicin administration upon cancer treatment (68).

However, our aim was to obtain candidate molecules with both target candidate profiles 1 and 5 (TCP-1, molecules that clear asexual blood stage parasitemia; TCP-5, molecules that block transmission by targeting the parasite), as desirable characteristics for candidate molecules (65, 66). Nonetheless, the findings herein described represent the initial steps of drug repurposing and drug discovery, characterized by the discovery of a new hit compound. Hit-to-lead optimization of the scaffold of epirubicin followed by structure-activity relationship studies (SAR) might lead to molecules with enhanced therapeutic indices with reduced toxicity and enhanced efficacy, oral bioavailability, and reasonable cost. SAR of doxorubicin have shown that the sugar moiety of anthracyclines (daunosamine) is toxicophoric group, i.e., essential for the drug’s cytotoxic effect (69). Moreover, blocking the amino function of the daunosamine sugar moiety might reduce toxicity, as it plays a crucial role in DNA binding (70). Conformational studies support the fact that daunosamine presents the most spatial flexibility among the anthracycline domains (71), tolerating modulation of its properties via sugar modifications (69). Examples of doxorubicin optimization have led to the development of expanded-spectrum anthracyclines like nemorubicin (72, 73) and sabarubicin (74), which are less cardiotoxic drugs. Based on these observations, we suggest alterations within the sugar moiety of epirubicin aiming to reduce its toxicity and generate compounds with improved pharmacological and pharmacokinetics profiles.

In conclusion, we have applied a computer-assisted chemogenomics drug repositioning workflow to identify new hits for malaria treatment. In doing so, we have demonstrated that epirubicin significantly blocks parasite development of sexual and asexual life stages in both P. falciparum and P. vivax, despite having been shown to present significant toxicity when used as an anticancer agent. Epirubicin was predicted to target *Plasmodium* GyrA. However, functional validation identified a second putative target in *Plasmodium*, a GPT enzyme involved in protein *N*-glycosylation. Both molecular targets predicted, either through computational or functional assays, are involved, in a broader perspective, in the metabolism of isoprenoids, a biological process essential to eukaryotic cells. Hence, epirubicin acts upon vital parasite functions, corroborating its antimalarial activity throughout the *Plasmodium* life cycle. Collectively, our findings encourage the use of chemogenomics repurposing strategies for interrogation of existing drug repositories against parasitic diseases as well as further investigation on other possible inhibitors of isoprenoid metabolism in the parasite. Future work can focus on epirubicin SAR and hit-to-lead optimization, aiming to develop new antimalarial drug candidates with enhanced therapeutic indices, reduced toxicity and enhanced efficacy, oral bioavailability, and reasonable cost.

## MATERIALS AND METHODS

### Repurposing of drugs from public databases.

Initially, we compiled a list of P. falciparum and P. vivax proteins with orthologues predicted to be absent in humans and rodents using the TDR Targets Database (75). Then, a sequence-based similarity search (E value, ≤10^−20^) was performed between *Plasmodium* proteins and all drug targets available in DrugBank (22) and TTD (23), according to methodology developed by Neves and coworkers (17).

Conserved amino acid residues are believed to perform important structural and/or functional roles in the protein. Herein, all the predicted *Plasmodium* protein targets were analyzed to determine if they share functional amino acid residues with their homologous human drug targets. Initially, predicted *Plasmodium* targets were aligned with their homologous drug targets using pairwise BLAST (76). Subsequently, functional regions among the approved drug targets and *Plasmodium* targets were compared using the ConSurf server (77, 78) using default parameters.

### Machine learning models.

Two data sets with phenotypic bioassay data for 3D7 (susceptible to chloroquine) (25–30) and W2 (resistant to chloroquine) (26, 28, 31–35) strains of P. falciparum were compiled from the PubChem BioAssay database (https://pubchem.ncbi.nlm.nih.gov/bioassay) (36, 37). The compounds with reproducible potency (IC_50_, ≤1 μM) were considered active, whereas the remaining compounds (IC_50_, >1 μM) were considered inactive, following the antiplasmodial cutoff of <1 μM for sensitive and resistant strains of *Plasmodium* spp., suggested by the Global Health Innovative Technology Fund (GHIT)-coordinated committee (79). All chemical structures and corresponding biological information were carefully curated according to the protocols proposed by Fourches and colleagues (38–40). Finally, a curated data set containing 2,853 active and 2,853 inactive compounds was used for modeling activity against the 3D7 sensitive strain, while a data set containing 3,086 active and 3,086 inactive compounds was used for modeling the W2 resistant strain.

The chemical structures of selected compounds were translated into Morgan fingerprints using the open-source cheminformatics toolkit RDKit v.2.4.0. (http://www.rdkit.org). Then, machine learning (ML) models were built separately for 3D7 and W2 data sets by combining these fingerprints with the GBM method (41). All ML classifiers were implemented using the R v.3.0.3.(80). The models were developed using the 5-fold external cross-validation procedure. Models were built using the modeling set, while the compounds in the momentary external set (fold) were employed for evaluation of predictive performance. Finally, the statistical performance of ML models was estimated using sensitivity (SE), specificity (SP), correct classification rate (CCR), and Cohen’s kappa coefficient (κ).

After modeling was complete, the most predictive models developed for 3D7 and W2 were employed for predicting the antiplasmodial activity of prioritized drugs. The reliability of predictions was estimated based on applicability domain, i.e., the Euclidean distances among each drug and the training set compounds used to develop ML models. If an investigated drug exceeded a distance threshold defined using default parameter Z at 0.5, the prediction was considered to be less trustworthy (81).

### *In vitro* assay against P. falciparum asexual stages.

P. falciparum strains 3D7 (drug sensitive), Dd2, and W2 (multidrug resistant) were cultured in RPMI 1640 medium (Sigma-Aldrich) supplemented with 10% human plasma (82). Synchronous cultures were obtained from treatment with a 5% d-sorbitol (Sigma-Aldrich) solution. Drug inhibition assays were performed by distributing ring-synchronized parasites (0.5% parasitemia and 2% hematocrit) in 96-well microplates in the presence of different concentrations of epirubicin (E9406; Sigma-Aldrich) in duplicate. Chloroquine was used as an antimalarial control. After 72 h of incubation, parasitemia was determined by fluorometry using Sybr green fluorescent dye, according to Hartwig et al. (83). EC_50_ values were calculated by plotting log dosing versus growth inhibition (expressed as percentage relative to drug-free control). Drugs tested were purchased from Sigma-Aldrich.

### Cytotoxicity assay in mammalian cell lines.

The cytotoxicity of epirubicin was assessed using the 3-(4,5-dimethyl-2-thiazolyl)-2,5-diphenyl-2H-tetrazolium bromide (MTT) assay to quantify the cell viability of two cell lines, namely, fibroblast-like cell line from monkey kidney tissue (COS-7) and human hepatoma (HepG2), via mitochondrial activity as previously described (84). Briefly, cells were cultivated at 5% CO_2_ and 37°C using Dulbecco’s modified Eagle medium supplemented with 10% heat-inactivated fetal bovine serum. Cells were seeded at a density of 10^5^ cells per well, and when the confluence was about 70%, the cells were incubated for 72 h with serial dilutions of epirubicin (200 to 0.097 μM) for COS-7 and (100 to 0.00038 μM) for HepG2 cells. Absorbance reading at an optical density at 570 nm (OD_570_) was done in a plate spectrophotometer, and the percent viability of cells was expressed as a percentage relative to an untreated control.

### *In vivo* activity on murine *Plasmodium* asexual stages.

The *in vivo* activity of epirubicin in a murine model of infection was evaluated in groups of 5 to 6 female C57BL/6JUnib mice (6 to 7 weeks old) obtained from the University of Campinas Animal Care Facility (no. 4574-1/2017 of the Ethics and Animal Utilization Committee at UNICAMP). The animals were housed in polypropylene cages in a pathogen-free animal facility at a temperature of 20 ± 3°C and relative humidity of 60% ± 5%, in a cycle of 12 h light/12 h darkness. At 3 h after intraperitoneal inoculation of 10^6^ GFP-expressing P. yoelii 17XNL-infected erythrocytes, animals were randomly divided into experimental groups and treatment was initiated (day 0). Epirubicin was administered intraperitoneally at 2, 4, and 6 mg/kg. The untreated control group received only the drug vehicle (10% DMSO). Treatment was administered daily until day 3 postinfection, following a modified Peter’s 4-day suppressive test (85). Parasitemia was measured by diluting 1 μl of whole blood obtained from mice tail in 100 μl of phosphate-buffered saline (PBS), followed by flow cytometry analysis using a 488-nm laser with 530/40 emission filters (FACSCalibur) (86). Antimalarial activity was expressed as a percentage of inhibition relative to the control group.

### Sample collection of P. vivax field isolates.

Due to the lack of a continuous *in vitro* culture, antimalarial assays in P. vivax require fresh blood samples to be performed. For this purpose, vivax malaria patients were recruited at Fundação de Medicina Tropical Doutor Heitor Vieira Dourado (FMT-HVD), Manaus, Amazonas, Brazil. A total of 10 ml of blood samples was collected from exclusively nonsevere vivax malaria-infected adult patients diagnosed by microscopy. Only patients with parasitemia higher than 1,000 parasites/μl and without any antimalarial treatment history in the last 60 days were included, after agreement to participate in the study by signing the Informed Consent Form. The project was approved by the Research Ethics Committee (no. 2.584969 of 6 April 2018).

### *Ex vivo* susceptibility against P. vivax clinical isolates.

The *ex vivo* susceptibility of P. vivax isolates was evaluated by the schizont maturation assay (87). For this assay, only blood samples containing more than 40% of ring parasites were used. Blood samples were centrifuged at 400 × *g*; the pellet was washed twice in McCoy 5A medium, passed through a cellulose column for leukocyte removal, and washed twice in medium. Parasitized erythrocyte suspension at 2% hematocrit was added to 96-well plates containing epirubicin (0.0095 to 5 μM) or chloroquine (0.00195 to 1 μM) in duplicate for each of the 10 serial dilutions, as well as untreated control wells. Parasites were cultured under hypoxic atmosphere conditions (5% CO_2_, 5% O_2_, and 90% N_2_) at 37°C for 35 to 50 h. The number of schizonts per 200 asexual parasites was determined for each drug concentration and control (87), and EC_50_ values were calculated using the online software ICestimator (http://www.antimalarial-icestimator.net/).

### Infection of Anopheles aquasalis by standard membrane-feeding assay.

*Anopheles aquasalis* mosquitoes used for this purpose were bred in the insectary of the Laboratory of Entomology at FMT-HVD, Manaus, Amazonas, Brazil, according to an established methodology (88). For *An. aquasalis* infection, blood from P. vivax-infected patients was centrifuged at 400 × *g* for 5 minutes, resuspended in nonimmune AB serum to a 40% hematocrit in the presence of epirubicin (5 or 10 μM) or the vehicle (0.1% DMSO) as a control, and given immediately to feed 100 adult females of *An. aquasalis* (3 to 5 days of age) per group. After 2 h of feeding, the fully engorged mosquitoes were transferred to a new cage with available 10% sugar solution in a room with controlled temperature (24 to 26°C) and humidity (70% to 80%) until dissection. At 7 days postinfection, the midguts from each experimental group were dissected in PBS, placed in a cover glass, stained with 0.1% merbromin, and analyzed by microscopy to determine the infection status. Infection rate was expressed as the percentage of mosquitoes with at least one oocyst, and the infection intensity was determined as the arithmetic mean of the oocysts counted per dissected midgut.

### *In vitro* inhibition of P. berghei ookinete formation by conversion assay.

The conversion assay evaluates a compound’s potential of inhibiting ookinete formation *in vitro* (89). Briefly, BALB/c mice were infected intraperitoneally with a mutant P. berghei line expressing luciferase (nLuc) under a ookinete-specific promoter. After 3 to 4 days, blood with circulating gametocytes was collected from mice and infected blood was incubated in 96-well plates with culture medium (RPMI 1640, 25 mM HEPES, 50 mg/liter hypoxanthine, and 1% penicillin/streptomycin/neomycin [PSN; pH 8.3]) containing a serial dilution of epirubicin. After 24 h of incubation at 21°C, a substrate for nLuc (Nano-Glo luciferase assay system, Promega) was added and plates were read in a luminometer. EC_50_ values were calculated by plotting log dosing versus growth inhibition (expressed as percentage relative to drug-free control).

### Chemical genomic profiling.

Chemical genomic profiling was performed with a Saccharomyces cerevisiae heterozygous yeast library for the ∼6,000 genes (catalog no. 95401.H4Pool; Invitrogen), in which single deletions are identified by molecular barcodes flanked by universal sequences (90). EC_20_ for epirubicin was determined as described previously (91). Briefly, the S. cerevisiae BY4743 wild-type strain (initial OD_600_ of 0.1) was incubated in 384-well plates in the presence of a serial dilution of epirubicin in yeast extract-peptone-dextrose (YPD) liquid medium (1% [wt/vol] yeast extract, 2% [wt/vol] peptone, and 2% [wt/vol] glucose) at 30°C with double orbital agitation of 200 rpm for 24 h. For competition assays, the library pool was inoculated (initial OD_600_ of 0.1) in YPD liquid medium in 48-well plates at 30°C with 200 rpm agitation in the presence of an EC_20_ concentration of epirubicin for 12 h (approximately 5 generations). The culture was diluted 1:20 for a further 12 hours of growth (∼10 generations). Every 5 generations, cells were harvested and pellets were collected for genomic DNA (gDNA) extraction with the Wizard genomic DNA purification kit (Promega). The upstream molecular barcodes were PCR amplified with U1 and U2 primers containing Illumina preadapters for multiplexing sequencing using the Illumina HiSeq 2500 platform from the University of São Paulo Genome Sequencing Center. Based on the barcode sequences, sequencing analysis was carried out by creating a “virtual genome,” to which reads were aligned and quantified per barcode. Heterozygous strains were ranked based on their epirubicin sensitivity after 10 generations. Strains most depleted within treated populations compared with an untreated control (top hits, *P* value of <0.001) were followed by gene ontology (GO) analysis.

### Homology modeling studies.

As there were no 3D crystallographic structures available of *Plasmodium* DNA gyrase or GlcNac-1-P-transferase (GPT) proteins at the time we conducted this research, they were constructed by homology modeling using the Swiss-Model server (92, 93). The primary sequences of each protein were obtained at the Uniprot server (94), and their FASTA sequences were submitted to Swiss-Model to obtain the homology models. The models that presented higher sequence identity and coverage with the templates were selected to be used in the refinement step. After modeling, we refined the structures using the GalaxyRefine server (95) and MolProbity server (45, 96, 97) to add hydrogen atoms and to analyze the quality statistics of the modeled proteins.

### Molecular docking calculations.

Docking calculations of epirubicin were performed using the Glide software (98) in extra precision (XP) mode. For DNA gyrase, we performed docking calculations of the protein with DNA strands (called gyrase-DNA) and without DNA strands (gyrase). We prepared the protein structures through the protein preparation Wizard tool (99, 100), following the standard protocol (101). The ligand structure was retrieved from PubChem (102) and prepared through the LigPrep tool (103), according to the tutorial. The protein grid coordinates were built based on ligands cocrystallized in the structures of homologue proteins, namely, levofloxacin (from PDB ID: 5EIX) for the DNA gyrase grid and tunicamycin (from PDB ID: 6BW5) for the GPT grid. The Visual Molecular Dynamics program (VMD) (104) was used for the visual inspection of 3D docking poses and to render the 3D molecular images.

### Statistical analysis.

Statistical analysis was performed using GraphPad Prism v.6 software. To calculate the EC_50_ in asexual and sexual blood stages of parasites and cell lines, a nonlinear regression curve was made with the data of the concentrations expressed in a logarithmic scale. The inhibition potential of chloroquine and epirubicin in P. vivax isolates was compared by the Mann-Whitney test. In the membrane feeding assay, infection rates between groups were compared using one-way ANOVA and Dunnet’s posttest. For *in vivo* data, group parasitemias were compared using ANOVA and Dunnet’s posttest. Data were considered statistically significant when the *P* value was <0.05.

## Supplementary Material

Supplemental file 1
