# Supplementary material for: Computational Chemogenomics Drug Repositioning Strategy Enables the Discovery of Epirubicin as a New Repurposed Hit for Plasmodium falciparum and P. vivax
Source: Antimicrob Agents Chemother. 2020 Aug 20;64(9):e02041-19. doi: 10.1128/AAC.02041-19 (PMC7449180; doi:10.1128/AAC.02041-19)
Supplement: Supplemental file 1 [file AAC.02041-19-s0001.pdf]

SUPPLEMENTARY MATERIAL

Table S1. List of approved drugs with predicted activity against human malaria parasites *P. falciparum* and/or *P. vivax*

**Drug**	**Original indication**	**Predicted *P. falciparum* and or *P. vivax* target(s)/ cellular component**
Sulindac	anti-inflammatory	PF3D7_1337100: protein kinase 6/cytoplasm<br>PF3D7_1014400: MO15-related protein kinase/cytoplasm
Isoprenaline	Bronchodilator and heart stimulant	PF3D7_1014400: MO15-related protein kinase/cytoplasm<br>PF3D7_1316000: protein kinase, putative/cytoplasm<br>PF3D7_1014400: MO15-related protein kinase/cytoplasm<br>PVX_122575: serine/threonine-protein kinase, putative/cytoplasm<br>PVX_118425: Ser/Thr protein kinase, putative/cytoplasm
Penciclovir	Antiviral	PF3D7_1037000: DNA polymerase zeta catalytic subunit, putative/membrane<br>PVX_110910: DNA polymerase zeta catalytic subunit, putative/membrane
Azelaic acid	Antineoplastic Agents, Dermatologic Agents	PF3D7_0625300: DNA polymerase 1, putative/no data<br>PVX_114370: DNA polymerase 1, putative/no data
Thiocarlide	Antibacterial	PF3D7_0511200: stearoyl-CoA delta 9 desaturase, putative/ endoplasmic reticulum
Epirubicin	Antibacterial, anticancer	PF3D7_1223300: DNA gyrase subunit A/apicoplast<br>PVX_123795: DNA gyrase subunit A/apicoplast
Levosimendan	Congestive heart failure	PF3D7_1446600: centrin-2/centrosome<br>PVX_090955: centrin-4, putative/centrosome
Nedocromil	Anti-Allergic	PF3D7_1443900: HSP90/apicoplast<br>PVX_118295: heat shock protein, putative/apicoplast

Besifloxacin	Opthalmic antibiotic	PF3D7_1223300: DNA gyrase subunit A/apicoplast<br>PVX_123795: DNA gyrase subunit A/apicoplast
Captopril	Hypertension	PF3D7_1311800: m1-family aminopeptidase/microneme, cytoplasm<br>PVX_122425: M1-family aminopeptidase, putative/microneme, cytoplasm
Mesalazine	Anti-inflammatory	PF3D7_1122800: calcium-dependent protein kinase, putative/no data<br>PF3D7_1316000: protein kinase, putative/no data<br>PVX_085300: calcium-dependent protein kinase, putative/no data<br>PVX_117985: protein kinase, putative/no data
Phenoxybenzamine	Anti- hypertensive	PF3D7_1446600: calmodulin

Data on drugs was retrieved from DrugBank [77] and target localization was obtained from PlasmoDB [95], according to Gene Ontology (GO)

Table S2. Quantification of parasitemia *in vivo* for epirubcin-treated and untreated mice.

	**Assay number**		**Epirubicin doses**		
		Nontreated	2 mg/kg	4 mg/kg	6 mg/kg
**D3**	#1	1,74	0,45	0,13	0,03
	#2	0,34	0,18	0,06	0
	#3	1,78	1,06	0,49	0,14
	Média parasitemia	**1,29**	**0,56**	**0,23**	**0,06**
	SD	0,82	0,45	0,23	0,07
	Inibição		**56,22**	**82,38**	**95,60**
**D5**	#1	1,72	1,38	1,06	0,39
	#2	1,32	1,29	0,36	0,11

	#3	2,05	3,95	2,69	0,63
	Média parasitemia	**1,70**	**2,21**	**1,37**	**0,38**
	SD	0,37	1,51	1,20	0,26
	Inibição		**0,00**	**19,25**	**77,80**
**D7**	#1	4,01	2,66	6,33	1,47
	#2	2,03	5,18	2,09	0,68
	#3	4,88	12,9	14,1	3,18
	Média parasitemia	**3,6**	**6,9**	**7,5**	**1,8**
	SD	1,46	5,34	6,09	1,28
	Inibição		**0,00**	**0,00**	**51,2**

Table S3. Chemical genomic profiling top hits identified upon Epirubicin treatment after 10 yeast generations.

ORF[a]	Gene	Log2 *fold change*[a]	p-value
YIR020C	-	-1,013800783	5,47E-05*
Ontology: unknown function			
YBR243C	*alg7*	-1,57836617	0,000255*
Ontology: UDP-N-acetyl-glucosamine-1-P transferase			
YOR038C	*hir2*	-1,019961612	0,000465*
Ontology: histone chaperone			
YBR285W	-	-0,957812381	0,000578*
Ontology: putative protein of unknown function			

[a] magnitude of the change in mean barcodes counts between treatment and control on the log2 scale

*p-value < 0,001

Table S4. Quality statistics for *Pf*DNA gyrase modeled protein obtained with Molprobity server

**Parameter**	**Goal**

All-atoms contacts	Clashscore§ (serious steric overlaps)	14.13 (53rd percentile)	-
Protein Geometry	Poor rotamers	0.62 %	< 0.3%
	Favored rotamers	98.14 %	> 98%
	Ramachandran outliers	0.39 %	< 0.05%
	Ramachandran favored	96.96 %	< 98 %
	MolProbity score*	1.83	-
	Cβ deviations > 0.25Å	3.96 %	0 %
	Bad bonds	0 %	0 %
	Bad angles	0.96 %	< 0.1 %
Peptide Omegas	Cis Prolines	0 %	≤ 5%

§100th clashscore percentile is the best among structures of comparable resolution, 0th percentile is the worst; *MolProbity score combines the clashscore, rotamer and Ramachandran evaluations into a single score, normalized to be on the same scale as X-ray resolution.

Table S5. Quality statistics for *Pf*GlcNac-1-P-transferase (GPT) modeled protein obtained with Molprobity server

	**Parameter**		**Goal**
All-atoms contacts	Clashscore§ (serious steric overlaps)	23.58 (23rd percentile)	-
Protein Geometry	Poor rotamers	1.08 %	< 0.3%
	Favored rotamers	97.37 %	> 98%
	Ramachandran outliers	1.01 %	< 0.05%
	Ramachandran favored	97.84 %	< 98 %
	MolProbity score*	1.93	-
	Cβ deviations > 0.25Å	4.53 %	0 %
	Bad bonds	0 %	0 %
	Bad angles	1.44 %	< 0.1 %

Peptide Omegas	Cis Prolines	0 %	≤ 5%

§100th clashscore percentile is the best among structures of comparable resolution, 0th percentile is the worst; *MolProbity score combines the clashscore, rotamer and Ramachandran evaluations into a single score, normalized to be on the same scale as X-ray resolution.

Supplementary Figures

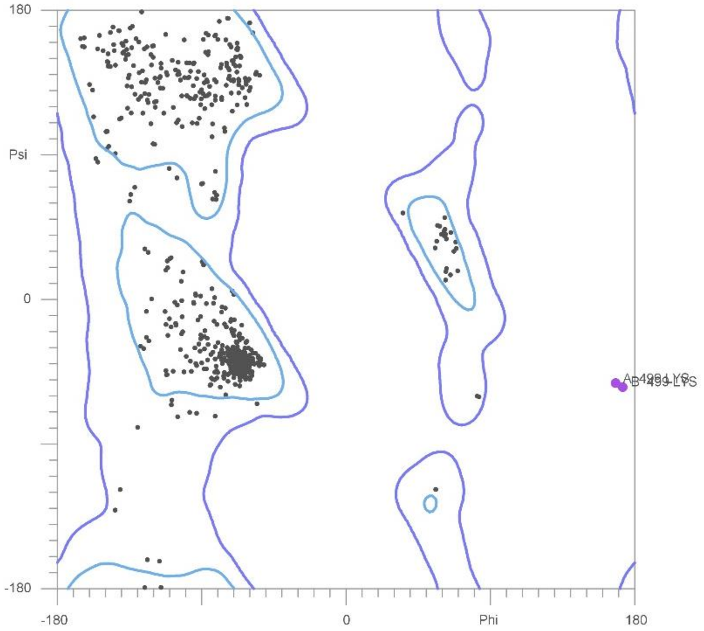

Figure S1. Ramachandran plot for *Pf*DNA gyrase modeled protein (allowed region in dark blue; favored region in cyano): 96.96% (989/1020) of all residues were in favored regions and 99.61% (1016/1020) of all residues were in allowed regions.

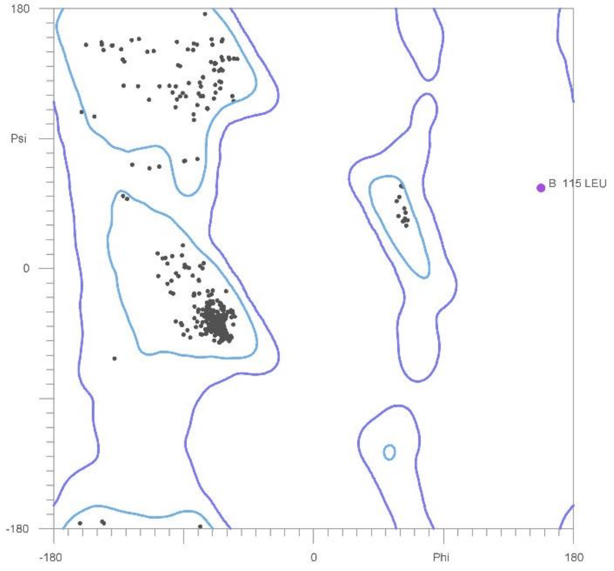

Figure S2. Ramachandran plot for *Pf*GlcNac-1-P-transferase (GPT) modeled protein (allowed region in dark blue; favored region in cyano): 97.84% (679/694) of all residues were in favored regions and 98.99% (687/694) of all residues were in allowed regions.

REFERENCES

1. World Health Organization. 2019. World Malaria Report 2019.
2. World Health Organization. 2016. World Malaria Report 2016.
3. World Health Organization. 2015. World Malaria Report 2015.
4. Paloque L, Witkowski B, Lelièvre J, Ouji M, Ben Haddou T, Ariey F, Robert A, Augereau JM, Ménard D, Meunier B, Benoit-Vical F. 2018. Endoperoxide-based compounds: cross-resistance with artemisinins and selection of a Plasmodium falciparum lineage with a K13 non-synonymous polymorphism. J Antimicrob Chemother 73:395–403.
5. Alexandre MA, Ferreira CO, Siqueira AM, Magalhães BL, Mourão MPG, Lacerda M V., Alecrim M das GC. 2010. Severe Plasmodium vivax malaria, Brazilian Amazon. Emerg Infect Dis 16:1611–1614.

6. Marques MM, Costa MRF, Santana Filho FS, Vieira JLF, Nascimento MTS, Brasil LW, Nogueira F, Silveira H, Reyes-Lecca RC, Monteiro WM, Lacerda MVG, Alecrim MGC. 2014. Plasmodium vivax chloroquine resistance and anemia in the western brazilian amazon. Antimicrob Agents Chemother 58:342–347.

7. Boyd M, Kitchen S. 1937. On the infectiousness of patients infected with Plasmodium vivax and Plasmodium falciparum. Am J Trop Med Hyg 17:253–62.

8. Nicola Nosengo. 2016. Can you teach old drugs new tricks? Nature 534:314–316.

9. Tan KR, Magill AJ, Parise ME, Arguin PM. 2011. Doxycycline for malaria chemoprophylaxis and treatment: Report from the CDC expert meeting on malaria chemoprophylaxis. Am J Trop Med Hyg 84:517–531.

10. Manyando C, Njunju EM, D'Alessandro U, Van geertruyden JP. 2013. Safety and Efficacy of Co-Trimoxazole for Treatment and Prevention of Plasmodium falciparum Malaria: A Systematic Review. PLoS One 8.

11. Andrews KT, Fisher G, Skinner-Adams TS. 2014. Drug repurposing and human parasitic protozoan diseases. Int J Parasitol Drugs Drug Resist 4:95–111.

12. Kundu CN, Das S, Nayak A, Satapathy SR, Das D, Siddharth S. 2015. Anti-malarials are anti-cancers and vice versa - One arrow two sparrows. Acta Trop 149:113–127.

13. Liu Z, Fang H, Reagan K, Xu X, Mendrick DL, Slikker W, Tong W. 2013. In silico drug repositioning: what we need to know. Drug Discov Today 18:110–5.

14. Andrade CH, Neves BJ, Melo-Filho CC, Rodrigues J, Silva DC, Braga RC, Cravo PVL. 2019. In Silico Chemogenomics Drug Repositioning Strategies for Neglected Tropical Diseases. Curr Med Chem 26:4355–4379.

15. Bispo NA, Culleton R, Silva LA, Cravo P. 2013. A Systematic In Silico Search for Target Similarity Identifies Several Approved Drugs with Potential Activity against the Plasmodium falciparum Apicoplast. PLoS One 8.

16. Lima MNN, Melo-Filho CC, Cassiano GC, Neves BJ, Alves VM, Braga RC, Cravo PVL, Muratov EN, Calit J, Bargieri DY, Costa FTM, Andrade CH. 2018. QSAR-Driven Design and Discovery of Novel Compounds With Antiplasmodial and Transmission Blocking Activities. Front

Pharmacol 9:146.

17. Neves BJ, Braga RC, Bezerra JCB, Cravo PVL, Andrade CH. 2015. In Silico Repositioning-Chemogenomics Strategy Identifies New Drugs with Potential Activity against Multiple Life Stages of Schistosoma mansoni. PLoS Negl Trop Dis 9.

18. De Oliveira AA, Neves BJ, Do Carmo Silva L, De Almeida Soares CM, Andrade CH, Pereira M. 2019. Drug repurposing for paracoccidioidomycosis through a computational chemogenomics framework. Front Microbiol 10.

19. Giuliani S, Silva AC, Borba JVVB, Ramos PIP, Paveley RA, Muratov EN, Andrade CH, Furnham N. 2018. Computationally-guided drug repurposing enables the discovery of kinase targets and inhibitors as new schistosomicidal agents. PLoS Comput Biol 14:e1006515.

20. Borba JVB, Silva AC, Ramos PIP, Grazzia N, Miguel DC, Muratov EN, Furnham N, Andrade CH. 2019. Unveiling the Kinomes of Leishmania infantum and L. braziliensis Empowers the Discovery of New Kinase Targets and Antileishmanial Compounds. Comput Struct Biotechnol J 17:352–361.

21. Earl H, Iddawela M. 2004. Epirubicin as adjuvant therapy in breast cancer. Expert Rev Anticancer Ther 4:189–195.

22. Magariños MP, Carmona SJ, Crowther GJ, Ralph SA, Roos DS, Shanmugam D, Van Voorhis WC, Agüero F. 2012. TDR targets: A chemogenomics resource for neglected diseases. Nucleic Acids Res 40:1118–1127.

23. Law V, Knox C, Djoumbou Y, Jewison T, Guo AC, Liu Y, MacIejewski A, Arndt D, Wilson M, Neveu V, Tang A, Gabriel G, Ly C, Adamjee S, Dame ZT, Han B, Zhou Y, Wishart DS. 2014. DrugBank 4.0: Shedding new light on drug metabolism. Nucleic Acids Res 42:1091–1097.

24. Li YH, Yu CY, Li XX, Zhang P, Tang J, Yang Q, Fu T, Zhang X, Cui X, Tu G, Zhang Y, Li S, Yang F, Sun Q, Qin C, Zeng X, Chen Z, Chen YZ, Zhu F. 2018. Therapeutic target database update 2018: enriched resource for facilitating bench-to-clinic research of targeted therapeutics. Nucleic Acids Res 46:D1121–D1127.

25. Agarwal P, States DJ. 1998. Comparative accuracy of methods for protein sequence similarity search. Bioinformatics 14:40–7.

26. Glaser F, Pupko T, Paz I, Bell RE, Bechor-Shental D, Martz E, Ben-Tal N. 2003. ConSurf: identification of functional regions in proteins by surface-mapping of phylogenetic information. Bioinformatics 19:163–4.

27. Ashkenazy H, Erez E, Martz E, Pupko T, Ben-Tal N. 2010. ConSurf 2010: calculating evolutionary conservation in sequence and structure of proteins and nucleic acids. Nucleic Acids Res 38:W529-33.

28. National Center for Biotechnology Information. PubChem BioAssay Database; AID=449703, Source=Genomics Institute of the Novartis Research Foundation, https://pubchem.ncbi.nlm.nih.gov/assay/assay.cgi?aid=449703 (accessed Jan. 22, 2017).

29. Plouffe D, Brinker A, McNamara C, Henson K, Kato N, Kuhen K, Nagle A, Adrián F, Matzen JT, Anderson P, Nam T, Gray NS, Chatterjee A, Janes J, Yan SF, Trager R, Caldwell JS, Schultz PG, Zhou Y, Winzeler EA. 2008. In silico activity profiling reveals the mechanism of action of antimalarials discovered in a high-throughput screen. Proc Natl Acad Sci 105:9059–9064.

30. National Center for Biotechnology Information. PubChem BioAssay Database; AID=524790, Source=National Institute of Allergy and Infectious Diseases, https://pubchem.ncbi.nlm.nih.gov/assay/assay.cgi?aid=524790 (accessed Jan. 22, 2017).

31. Yuan J, Johnson RL, Huang R, Wichterman J, Jiang H, Hayton K, Fidock DA, Wellems TE, Inglese J, Austin CP, Su X. 2009. Genetic mapping of targets mediating differential chemical phenotypes in Plasmodium falciparum. Nat Chem Biol 5:765–771.

32. National Center for Biotechnology Information. PubChem BioAssay Database; AID=1828, Source=NIH Chemical Genomics Center, https://pubchem.ncbi.nlm.nih.gov/assay/assay.cgi?aid=1828 (accessed Jan. 22, 2017).

33. National Center for Biotechnology Information. PubChem BioAssay Database; AID=660866, Source=Medicines for Malaria Venture, https://pubchem.ncbi.nlm.nih.gov/assay/assay.cgi?aid=660866 (accessed Jan. 22, 2017).

34. National Center for Biotechnology Information. PubChem BioAssay Database; AID=449704, Source= Genomics Institute of the Novartis Research Foundation, https://pubchem.ncbi.nlm.nih.gov/assay/assay.cgi?aid=449704 (accessed Jan. 22, 2017).

35. National Center for Biotechnology Information. PubChem BioAssay Database; AID= 524796,

Source=National Institute of Allergy and Infectious Diseases, https://pubchem.ncbi.nlm.nih.gov/assay/assay.cgi?aid=524796 (accessed Jan. 22, 2017).

36. National Center for Biotechnology Information. PubChem BioAssay Database; AID= 1883, Source=NIH Chemical Genomics Center, https://pubchem.ncbi.nlm.nih.gov/assay/assay.cgi?aid=1883 (accessed Jan. 22, 2017).

37. Cross RM, Maignan JR, Mutka TS, Luong L, Sargent J, Kyle DE, Manetsch R. 2011. Optimization of 1,2,3,4-Tetrahydroacridin-9(10 H)-ones as Antimalarials Utilizing Structure–Activity and Structure–Property Relationships. J Med Chem 54:4399–4426.

38. National Center for Biotechnology Information. PubChem BioAssay Database; AID= 606570, Source=NIH University of South Florida, https://pubchem.ncbi.nlm.nih.gov/assay/assay.cgi?aid=606570 (accessed Jan. 22, 2017).

39. Wang Y, Xiao J, Suzek TO, Zhang J, Wang J, Zhou Z, Han L, Karapetyan K, Dracheva S, Shoemaker BA, Bolton E, Gindulyte A, Bryant SH. 2012. PubChem's BioAssay Database. Nucleic Acids Res 40:D400–D412.

40. Wang Y, Bolton E, Dracheva S, Karapetyan K, Shoemaker BA, Suzek TO, Wang J, Xiao J, Zhang J, Bryant SH. 2010. An overview of the PubChem BioAssay resource. Nucleic Acids Res 38:D255-66.

41. Katsuno K, Burrows JN, Duncan K, Huijsduijnen RH Van, Kaneko T, Kita K, Mowbray CE, Schmatz D, Warner P, Slingsby BT. 2015. Hit and lead criteria in drug discovery for infectious diseases of the developing world. Nat Publ Gr 1–8.

42. Fourches D, Muratov E, Tropsha A. 2010. Trust, but verify: on the importance of chemical structure curation in cheminformatics and QSAR modeling research. J Chem Inf Model 50:1189–204.

43. Fourches D, Muratov E, Tropsha A. 2015. Curation of chemogenomics data. Nat Chem Biol 11:535–535.

44. Fourches D, Muratov E, Tropsha A. 2016. Trust, but Verify II: A Practical Guide to Chemogenomics Data Curation. J Chem Inf Model 56:1243–1252.

45. Friedman JH. 2001. Greedy Function Approximation: A Gradient Boosting Machine. Ann Stat

29:1189–1232.

46. R Development Core Team. 2008. R: A Language and Environment for Statistical Computing. R Foundation for Statistical Computing. R Foundation for Statistical Computing, Vienna, Austria.

47. Tropsha A, Golbraikh A. 2007. Predictive QSAR modeling workflow, model applicability domains, and virtual screening. Curr Pharm Des 13:3494–504.

48. Trager W, Jensen JB. 1976. Human malaria parasites in continuous culture. Science 193:673–5.

49. Russell B, Malleret B, Suwanarusk R, Anthony C, Kanlaya S, Lau YL, Woodrow CJ, Nosten F, Renia L. 2013. Field-based flow cytometry for ex vivo characterization of plasmodium vivax and P. falciparum antimalarial sensitivity. Antimicrob Agents Chemother 57:5170–5174.

50. Kumar P, Nagarajan A, Uchil PD. 2018. Analysis of cell viability by the MTT assay. Cold Spring Harb Protoc 469–471.

51. Peters W. 1975. The chemotherapy of rodent malaria, XXII. The value of drug-resistant strains of P. berghei in screening for blood schizontocidal activity. Ann Trop Med Parasitol 69:155–171.

52. Ono T, Tadakuma T, Rodriguez A. 2007. Plasmodium yoelii yoelii 17XNL constitutively expressing GFP throughout the life cycle. Exp Parasitol 115:310–313.

53. Russell BM, Udomsangpetch R, Rieckmann KH, Kotecka BM, Coleman RE, Sattabongkot J. 2003. Simple In Vitro Assay for Determining the Sensitivity of Plasmodium vivax Isolates from Fresh Human Blood to Antimalarials in Areas where P . vivax Is Endemic. Antimicrob Agents Chemother 47:170–173.

54. Pimenta PFP, Orfano AS, Bahia AC, Duarte APM, Ríos-velásquez CM, Melo FF, Pessoa FAC, Oliveira GA, Campos KMM, Villegas LM, Rodrigues NB, Nacif-pimenta R, Simões RC, Monteiro WM, Amino R, Traub-cseko YM, Lima JBP, Barbosa MG V, Lacerda MVG, Tadei WP, Secundino NFC. 2015. An overview of malaria transmission from the perspective of Amazon Anopheles vectors. Mem Inst Oswaldo Cruz 110:23–47.

55. Calit J, Drobescu I, Gaitán XA, Borges MH, Ramos MS, Eastman RT, Bargieri DY. 2018. Screening the Pathogen Box against Plasmodium sexual stages using a new nanoluciferase based transgenic line of P. berghei identifies transmission-blocking compounds. Antimicrob Agents Chemother.

56. Giaever G, Shoemaker DD, Jones TW, Liang H, Winzeler EA, Astromoff A, Davis RW. 1999. Genomic profiling of drug sensitivities via induced haploinsufficiency. Nat Genet 21:278–283.

57. Hoepfner D, Helliwell SB, Sadlish H, Schuierer S, Filipuzzi I, Brachat S, Bhullar B, Plikat U, Abraham Y, Altorfer M, Aust T, Baeriswyl L, Cerino R, Chang L, Estoppey D, Eichenberger J, Frederiksen M, Hartmann N, Hohendahl A, Knapp B, Krastel P, Melin N, Nigsch F, Oakeley EJ, Petitjean V, Petersen F, Riedl R, Schmitt EK, Staedtler F, Studer C, Tallarico JA, Wetzel S, Fishman MC, Porter JA, Movva NR. 2014. High-resolution chemical dissection of a model eukaryote reveals targets, pathways and gene functions. Microbiol Res 169:107–120.

58. Biasini M, Bienert S, Waterhouse A, Arnold K, Studer G, Schmidt T, Kiefer F, Cassarino TG, Bertoni M, Bordoli L, Schwede T. 2014. SWISS-MODEL: Modelling protein tertiary and quaternary structure using evolutionary information. Nucleic Acids Res 42:1–7.

59. Waterhouse A, Bertoni M, Bienert S, Studer G, Tauriello G, Gumienny R, Heer FT, De Beer TAP, Rempfer C, Bordoli L, Lepore R, Schwede T. 2018. SWISS-MODEL: Homology modelling of protein structures and complexes. Nucleic Acids Res 46:W296–W303.

60. Bateman A. 2019. UniProt: A worldwide hub of protein knowledge. Nucleic Acids Res 47:D506–D515.

61. Ko J, Park H, Heo L, Seok C. 2012. GalaxyWEB server for protein structure prediction and refinement. Nucleic Acids Res 40:294–297.

62. Hintze BJ, Lewis SM, Richardson JS, Richardson DC. 2016. Molprobity's ultimate rotamer-library distributions for model validation. Proteins Struct Funct Bioinforma 84:1177–1189.

63. Davis IW, Leaver-Fay A, Chen VB, Block JN, Kapral GJ, Wang X, Murray LW, Arendall WB, Snoeyink J, Richardson JS, Richardson DC. 2007. MolProbity: All-atom contacts and structure validation for proteins and nucleic acids. Nucleic Acids Res 35:375–383.

64. Chen VB, Arendall WB, Headd JJ, Keedy DA, Immormino RM, Kapral GJ, Murray LW, Richardson JS, Richardson DC. 2010. MolProbity: All-atom structure validation for macromolecular crystallography. Acta Crystallogr Sect D Biol Crystallogr 66:12–21.

65. Friesner RA, Murphy RB, Repasky MP, Frye LL, Greenwood JR, Halgren TA, Sanschagrin PC, Mainz DT. 2006. Extra precision glide: Docking and scoring incorporating a model of hydrophobic enclosure for protein-ligand complexes. J Med Chem 49:6177–6196.

66. Madhavi Sastry G, Adzhigirey M, Day T, Annabhimoju R, Sherman W. 2013. Protein and ligand preparation: Parameters, protocols, and influence on virtual screening enrichments. J Comput Aided Mol Des 27:221–234.

67. Schrödinger Release 2015-2: Protein Preparation Wizard, Schrödinger, LLC, New York, NY, 2015.

68. Banks JL, Beard HS, Cao Y, Cho AE, Damm W, Farid R, Felts AK, Halgren TA, Mainz DT, Maple JR, Murphy R, Philipp DM, Repasky MP, Zhang LY, Berne BJ, Friesner RA, Gallicchio E, Levy RM. 2005. Integrated Modeling Program, Applied Chemical Theory (IMPACT). J Comput Chem 26:1752–1780.

69. Kim S, Thiessen PA, Bolton EE, Chen J, Fu G, Gindulyte A, Han L, He J, He S, Shoemaker BA, Wang J, Yu B, Zhang J, Bryant SH. 2016. PubChem substance and compound databases. Nucleic Acids Res 44:D1202–D1213.

70. Schrödinger Release 2015-2: LigPrep, Schrödinger, LLC, New York, NY, 2015.

71. Humphrey W, Dalke A, Schulten K. 1996. VDM: Visual Molecular Dynamics. J Mol Graph 7855:33–38.

72. Andrade CH, Neves BJ, Melo-Filho CC, Rodrigues J, Silva DC, Braga RC, Cravo PVL. 2018. In Silico Chemogenomics Drug Repositioning Strategies for Neglected Tropical Diseases. Curr Med Chem 26:1–22.

73. Silva LA, Vinaud MC, Castro AM, Cravo PVL, Bezerra JCB. 2015. In silico search of energy metabolism inhibitors for alternative leishmaniasis treatments. Biomed Res Int 2015.

74. Fourches D, Muratov E, Tropsha A. 2010. Trust, But Verify: On the Importance of Chemical Structure Curation in Cheminformatics and QSAR Modeling Research. J Chem Inf Model 50:1189–1204.

75. Zhu X, Lehrman MA. 1990. Cloning, sequence and expression of cDNA encoding Hamster UDP-GlcNAc:dolichol phosphate N-acetylglucosamine-1-phosphate transferase. J Biol Chem 265:14250–14255.

76. Imlay L, Odom AR. 2014. Isoprenoid Metabolism in Apicomplexan Parasites. Curr Clin Microbiol Reports 1:37–50.

77. Veselkov DA, Laponogov I, Pan X-S, Selvarajah J, Skamrova GB, Branstrom A, Narasimhan J, Prasad JVNV, Fisher LM, Sanderson MR. 2016. Structure of a quinolone-stabilized cleavage complex of topoisomerase IV from Klebsiella pneumoniae and comparison with a related Streptococcus pneumoniae complex . Acta Crystallogr Sect D Struct Biol 72:488–496.

78. Yoo J, Mashalidis EH, Kuk ACY, Yamamoto K, Kaeser B, Ichikawa S, Lee SY. 2018. GlcNAc-1-P-transferase-tunicamycin complex structure reveals basis for inhibition of N-glycosylation. Nat Struct Mol Biol 25:217–224.

79. Mogire R, Akala H, Macharia R, Juma D, Cheruiyot A, Andagalu B, Brown M, El-Shemy H, Nyanjom S. 2017. Target-similarity search using Plasmodium falciparum proteome identifies approved drugs with anti-malarial activity and their possible targets. PLoS One 12:1–24.

80. Knox C, Law V, Jewison T, Liu P, Ly S, Frolkis A, Pon A, Banco K, Mak C, Neveu V, Djoumbou Y, Eisner R, Guo AC, Wishart DS. 2011. DrugBank 3.0: A comprehensive resource for “Omics” research on drugs. Nucleic Acids Res 39.

81. e Silva LFR, de Magalhães PM, Costa MRF, Alecrim M das GC, Chaves FCM, Hidalgo A de F, Pohlit AM, Vieira PPR. 2012. In vitro susceptibility of Plasmodium falciparum Welch field isolates to infusions prepared from Artemisia annua L. cultivated in the Brazilian Amazon. Mem Inst Oswaldo Cruz 107:859–866.

82. Cravo P, Culleton R, Afonso A, Ferreira I, do Rosario V. 2006. Mechanisms of Drug Resistance in Malaria: Current and New Challenges. Antiinfect Agents Med Chem 5:63–73.

83. Warhurst DC, Killick-Kendrick R. 1967. Spontaneous Resistance to Chloroquine in a Strain of Rodent Malaria (Plasmodium berghei yoelii). Nature 44–47.

84. Filho FS de S, Arcanjo AR de L, Chehuan YM, Costa MR, Martinez-Espinosa FE, Vieira JL, Barbosa M das GV, Alecrim WD, Alecrim M das GC. 2007. Chloroquine- Resistant Plasmodium vivax, Brazilian Amazon. Emerg Infect Dis 13:1125–1126.

85. Coatney GR. 1963. Pitfalls in a Discovery: The Chronicle of Chloroquine. Am J Med Hyigiene 12:121–128.

86. Price RN, Douglas NM, Anstey NM. 2009. New developments in Plasmodium vivax malaria: Severe disease and the rise of chloroquine resistance. Curr Opin Infect Dis 22:430–435.

87. Mueller I, Galinski MR, Baird JK, Carlton JM, Kochar DK, Alonso PL, del Portillo HA. 2009. Key gaps in the knowledge of Plasmodium vivax, a neglected human malaria parasite. Lancet Infect Dis 9:555–566.

88. Arisue N, Hashimoto T. 2015. Phylogeny and evolution of apicoplasts and apicomplexan parasites. Parasitol Int 64:254–259.

89. Lynn R, Giaever G, Swanberg SL, Wang JC. 1986. Tandem regions of yeast DNA Topoisomerase II share homology with different subunits of bacterial gyrase. Science (80-) 233:647–649.

90. Samuelson J, Banerjee S, Magnelli P, Cui J, Kelleher DJ, Gilmore R, Robbins PW. 2005. The diversity of dolichol-linked precursors to Asn-linked glycans likely results from secondary loss of sets of glycosyltransferases. Proc Natl Acad Sci 102:1548–1553.

91. Barnes G, Hansen WJ, Holcomb CL, Rine J. 1984. Asparagine-linked glycosylation in Saccharomyces cerevisiae: genetic analysis of an early step. Mol Cell Biol 4:2381–2388.

92. Robert V, Bourgouin C, Depoix D, Thouvenot C, Lombard M-N, Grellier P. 2008. Malaria and obesity: obese mice are resistant to cerebral malaria. Malar J 7:81.

93. Lombard J. 2016. The multiple evolutionary origins of the eukaryotic N-glycosylation pathway. Biol Direct 11.

94. Naik RS, Venkatesan M, Gowda DC. 2001. Plasmodium falciparum: The Lethal Effects of Tunicamycin and Mevastatin on the Parasite Are Not Mediated by the Inhibition of N-Linked Oligosaccharide Biosynthesis. Exp Parasitol 98:110–114.

95. Yeh E, DeRisi JL. 2011. Chemical rescue of malaria parasites lacking an apicoplast defines organelle function in blood-stage plasmodium falciparum. PLoS Biol 9.

96. Yuan G, Regel I, Lian F, Friedrich T, Hitkova I, Hofheinz RD, Ströbel P, Langer R, Keller G, Röcken C, Zimmermann W, Schmid RM, Ebert MPA, Burgermeister E. 2013. WNT6 is a novel target gene of caveolin-1 promoting chemoresistance to epirubicin in human gastric cancer cells. Oncogene 32:375–387.

97. Burrows JN, Huijsduijnen RH Van, Möhrle JJ, Oeuvray C, Wells TNC. 2013. Designing the next generation of medicines for malaria control and eradication. Malar J 12:1–20.

98. Burrows JN, Duparc S, Gutteridge WE, Hooft van Huijsduijnen R, Kaszubska W, Macintyre F,

Mazzuri S, Möhrle JJ, Wells TNC. 2017. New developments in anti-malarial target candidate and product profiles. Malar J 16:26.

99. Plosker GL FD. 1993. Epirubicin - A review of its pharmacodynamic and pharmacokinetic properties,and therapeutic use in cancer chemotherapy. Drugs1 45:788–856.

100. Alderton PM, Gross J, Green MD. 1992. Comparative Study of Doxorubicin, Mitoxantrone, and Epirubicin in Combination with ICRF-187 (ADR-529) in a Chronic Cardiotoxicity Animal Model. Cancer Res 52:194–201.

101. Zunino F, Pratesi G, Perego P. 2001. Role of the sugar moiety in the pharmacological activity of anthracyclines : development of a novel series of disaccharide analogs 61:933–938.

102. Zunino F, Di Marco A, Zaccara A, Gambetta RA. 1980. The interaction of daunorubicin and doxorubicin with DNA and chromatin. Biochim Biophys Acta 607:206–214.

103. Zhu S, Yan L, Ji X, Lu W. 2010. Conformational diversity of anthracycline anticancer antibiotics: A density functional theory calculation. J Mol Struct THEOCHEM 951:60–68.

104. Ripamonti M, Capolongo L, Melegaro G, Gornati C, Bargiotti A, Caruso M, Grandi M, Suarato A. 1996. Morpholinylanthracyclines: Cytotoxicity and antitumor activity of differently modified derivatives. Invest New Drugs 14:139–146.

105. Broggini M. 2008. Nemorubicin. Top Curr Chem 283:191–206.

106. Menna P, Minotti G, Salvatorelli E. 2007. In vitro modeling of the structure-activity determinants of anthracycline cardiotoxicity. Cell Biol Toxicol 23:49–62
